# Investigation of a robust pretreatment technique based on ultrasound-assisted, cost-effective ionic liquid for enhancing saccharification and bioethanol production from wheat straw

**DOI:** 10.1038/s41598-022-27258-9

**Published:** 2023-01-09

**Authors:** Zhila Ziaei-Rad, Mohammad Pazouki, Jamshid Fooladi, Mehrdad Azin, Sathyanarayana N. Gummadi, Abdollah Allahverdi

**Affiliations:** 1 Department of Biotechnology, Faculty of Biological Science, Alzahra University, Tehran, Iran; 2 Department of Energy, Materials and Energy Research Center, Karaj, Iran; 3 Department of Biotechnology, Iranian Research Organization for Science & Technology, Tehran, Iran; 4 Department of Biotechnology, BJM School of Biosciences, Indian Institute of Technology Madras, Chennai, 600 036 India; 5 Department of Biophysics, Faculty of Biological Science, Tarbiat Modares University, Tehran, 14115-154 Iran

**Keywords:** Environmental biotechnology, Environmental microbiology

## Abstract

Application of cost-effective pretreatment of wheat straw is an important stage for massive bioethanol production. A new approach is aimed to enhance the pretreatment of wheat straw by using low-cost ionic liquid [TEA][HSO_4_] coupled with ultrasound irradiation. The pretreatment was conducted both at room temperature and at 130 °C with a high biomass loading rate of 20% and 20% wt water assisted by ultrasound at 100 W-24 kHz for 15 and 30 min. Wheat straw pretreated at 130 °C for 15 and 30 min had high delignification rates of 67.8% and 74.9%, respectively, and hemicellulose removal rates of 47.0% and 52.2%. Moreover, this pretreatment resulted in producing total reducing sugars of 24.5 and 32.1 mg/mL in enzymatic saccharification, respectively, which corresponds to saccharification yields of 67.7% and 79.8% with commercial cellulase enzyme CelluMax for 72 h. The ethanol generation rates of 38.9 and 42.0 g/L were attained for pretreated samples for 15 and 30 min, equivalent to the yields of 76.1% and 82.2% of the maximum theoretical yield following 48 h of fermentation. This demonstration provided a cheap and promising pretreatment technology in terms of efficiency and shortening the pretreatment time based on applying low-cost ionic liquid and efficient ultrasound pretreatment techniques, which facilitated the feasibility of this approach and could further develop the future of biorefinery.

## Introduction

Lignocellulosic biomass, as well as agriculture by-products, forest residues, energy crops, municipal solid waste, and different materials, are widely available bioresources on Earth. Biorefineries can utilize these resources as some kind of feedstock so that oil refineries may turn them into sources of fuels, platform chemicals, or other products^1–3^. Platform chemicals are small chemical molecules used in various downstream processes. Platform chemicals derived from lignocellulose biomass can be classified into six groups based on the number of carbon atoms in the molecule. One platform chemical from each group is mentioned: methanol (1 C), ethanol (2 C), lactic acid (3 C), butanol (4 C), furfural (5 C), and sorbitol (6 C)^4^. In this regard, a significant quantity of waste is generated by agro-based industries as by-products, consisting primarily of lignocellulosic biomass^5^. Lignocellulosic biomass is characterized by renewability and prevalence as well as its role in the extensive and inexpensive generation of bio-energy and high-value fine chemicals, which has drawn huge academic interest^6,7^. Wheat straw is a major agricultural by-product that is annually produced in Iran and contains cellulose (33–38%), hemicellulose (26–32%), lignin (17–19%), and ash (6–8%)^1^.

Lignocellulosic biomass comprises biopolymers of cellulose and hemicellulose carbohydrates in conjunction with lignin. Cellulose is a crystalline linear polysaccharide comprised of D-glucose subunits that are bound to each other by *β-*(1, 4)-glycosidic bonds, a substrate that produces bioethanol when fermented. However, short-chain amorphous hemicellulose hydrolysis produces different fermentable saccharide monomers due to its structural components. Hemicelluloses represent a varied polysaccharide group with the β-(1,4) bonds form of pentose (C5) and hexose (C6) sugars. Hemicelluloses increase the resilience and rigidity of plant cell walls due to the embedding and interaction of cellulose and lignin^8^. Lignin, an aromatic polymer with high branches, binds tightly to hemicellulose and cellulose fibers and it prevents the accessibility of cellulolytic enzymes to polysaccharide components^1–3^. Fermentable sugars are quite difficult to generate due to covalent cross-linking and the stiff morphology of lignocellulosic biomass^2,3,9–11^. Thus, this structure can be disrupted through pretreatment to ensure a freely accessible surface for enzymatic hydrolysis. Given that lignocellulosic biomass pretreatment significantly influences the effectiveness of saccharification and fermentation processes, it plays a role and is a key step in biofuel production^1,2,9,10,12^.

The severe reaction conditions, toxicity, and high energy consumption of conventional acid and alkali treatments have redirected the focus of recent research to alternative methods^13^. Ionic Liquids (ILs), regarded as organic salts, comprise small inorganic anions and large organic cations, and they are employed for cellulose fractionation and lignocellulosic biomass pretreatment^14,15^. By choosing appropriate anion and alkyl constituents of the cation, the characteristics of solvents are manipulated and used broadly in many fields^16^. ILs are famous as environmentally friendly solvents due to such properties as high chemical and thermal stability, non-flammability, no volatility, and relative non-toxicity^17,18^. In ILs, a hydrogen bond forms between non-hydrated anions and sugar hydroxyl protons, which helps ILs dissolve lignin and carbohydrates simultaneously^9^. Pretreatment of the biomass with ILs offers several advantages, including low processing pressure, thus reducing friction-abrasion and the separation of new products^19^.

A new generation of ILs called “low-cost ILs” has evolved. They are competitive with respect to conventional ILs. These new ILs are cheaper and more environmentally friendly, possess better thermal stability, and tolerate much more water^19–23^. In previous studies, it has been demonstrated that the addition of 10–40% water to ionoSolv ILs is needed for effective fractionation to occur. This has been attributed to several effects, including the necessity of water for the hydrolysis reactions that are required to separate the components from each other, to prevent sulfation reactions between hydrogen sulfate and hydroxyl groups in the biomass, and finally to reduce the viscosity of the solvent^21^.

Low-cost ILs are easy to synthesize and can be produced in the laboratory through the neutralization of mineral acids by organic amines without the need for any further purification. This is the reason why protic ILs such as triethylammonium hydrogen sulfate [TEA][HSO_4_], have drawn much attention in the published literature for economic reasons^20–22^. The cost of bulk-scale low-cost ILs has been reduced to $1.2 per kilogram, which is a major breakthrough in the economic feasibility of *IonoSolv* production. Another advantage of using *IonoSolv* in the delignification of biomass is the tolerability of the IL in the presence of a considerable amount of water, which has a notable effect on the cost-effectiveness of the process. It is worth noting that triethylammonium hydrogen sulfate [TEA][HSO4] work under ~ 20% (w/w) water hydrous conditions^21,22^. Further, besides the use of inexpensive ILs with a considerable amount of water, biomass loading is utilized, being an important and economically justified parameter for the process. The acceptable rate of biomass loading is 10% in the literature^24–26^. Thus, the application of a high biomass load that gives rise to greater possibilities for the industrial pretreatment process should be taken into consideration^27^.

Regarding the purpose of boosting the cellulose dissolution in IL, a number of studies have considered employing ultrasonic techniques coupled with IL pretreatment^28–31^.

Ultrasound (US) instruments emit mechanical-acoustic waves with frequencies ranging from 20 kHz to 500 MHz^32^. The properties of these waves help improve several chemical reactions through the mixing of suspensions to assist mass transfer. Furthermore, these waves cause the implosion of microbubbles at high pressure and temperature, which accelerates the reactions. The application of US with a frequency of 20–50 kHz has been reported to prompt porosity in the cellulose backbone and induce rupture in the α-O-4 or β-O-4 bond-linkage in lignin, which causes lignin separation from the lignocellulosic matrix^33–35^.

Improvements in dissolution and fractionation of lignocellulosic biomass were reported through various ultrasound-assisted pretreatment techniques, such as dilute acid, alkaline solutions, organic solvents, and ILs^35,36^. In recent years, ultrasound-assisted IL pretreatment has been broadly studied, and it offers great opportunities for enhancing enzymatic hydrolysis yield while increasing conversion rate and shortening the pretreatment time^29,31,37^.

The combination of two pretreatment processes leads to an increase in the fermentable sugar concentration compared to that in a single pretreatment process^38^.

Even if IL has lower viscosity, treatment of lignocellulose with ILs induces a high-viscous pulp, when the concentration of biomass reaches 5–10% (w/w) This high viscosity prevents infiltration of IL into the lignocellulose matrix even at higher temperatures and times (120 °C, 2 h). Even the use of ultrasonic pretreatment alone does not have a positive effect on enzymatic hydrolysis. Pretreatment itself on the cellulose saccharification ratio indicates that the increase in porosity of cellulose fiber by extreme shear stress (physical effect) and lignin oxidation by OH radicals (chemical effect) was not sufficient to enhance enzymatic hydrolysis under the examined conditions. Therefore, a combination of ultrasonic and IL treatments synergistically reduces the viscosity and therefore enhances enzymatic hydrolysis^33^. In another way, we can point out that heating alone is less effective than ultrasound, and that is less effective than a combination of ultrasound and IL when treating lignocellulosic biomass in terms of viscosity^39^. Therefore, the ultrasonic-assisted IL pretreatment process is a positive and effective substitute for the traditional pretreatment method for lignocellulosic biomass^28^.

Nevertheless, a combination of low-cost IL and ultrasonication for pretreatment of agricultural residue has not been investigated so far. The majority of aspects investigated in this work have not been previously considered by any other study. Examples are the combination of applying low-cost IL with ultrasound to pretreat wheat straw at high biomass loading (1:5 g/g as opposed to 1:10 g/g, which is insufficient for economical biofuel production) and shortening the pretreatment time. The combination of higher biomass loading and much shorter pretreatment would greatly decrease pretreatment reactor size and hence capital expenditure, leading to a significantly intensified process. The addition of water to ionoSolv ILs is required for effective fractionation to occur. Following that, pretreatment with [TEA][HSO4] was carried out under ~ 20% (w/w) hydrous conditions. Furthermore, this study targets analyzing the impact of temperature on the dissolution efficiency of wheat straw by applying a comparative study of ultrasound heat [TEA][HSO_4_] with ultrasound [TEA][HSO_4_], as well as applying commercial cellulase from *Trichoderma reesei* in enzymatic hydrolysis, and bioethanol recovery for the ionoSolv pretreatment in general. As far as we are concerned, the literature on cost-effective IL remains limited to the saccharification step, and no comprehensive corresponding process has yet been addressed.

## Materials and methods

### Lignocellulosic biomass and chemical reagents (materials)

Wheat straw, which was harvested in 2018, was procured from Aradan (Semnan, Iran). It was rinsed, dried at a temperature of 50 °C, milled to a size less than 0.6 mm (< 30 on a mesh scale), and kept in a desiccator before its treatment. Being an inexpensive ionic liquid, [TEA][HSO_4_] is a product of the synthesis of sulfuric acid (750 mmol) and triethylamine (750 mmol). It was dried at a rotary evaporator for 2 h and freeze-dried for 72 h^19–21^. This ionic liquid was retrieved in the form of a white, hygroscopic solid and maintained in a desiccator. Nuclear Magnetic Resonance (NMR) was employed to characterize the synthesized IL to verify the IL’s structural composition. A Bruker UXNMR spectrometer was utilized to record NMR at a 500 MHz frequency for ^1^H NMR and 126 MHz for ^13^C NMR with a 5 mm QNP probe. For the solvent, D_2_O was employed.

The cellulase (CelluMax GFL- Novozymes) produced by *Trichoderma reesei* was purchased from Turkey. It contained cellulase, xylanase, hemicellulase and beta-glucanase complexes (E.C 3.2.1.4), (E.C.3.2.1.8), and (E.C.3.2.1.6) with 56 FPU/mL activity determined by the NREL method^40^. All materials and chemical substances were provided by Merck and/or Sigma-Aldrich.

### Pretreatment with ionic liquid and ultrasound

A 1:5 g/g biomass-to-solvent loading was prepared by mixing 2 g of wheat straw, 8 g of [TEA][HSO_4_], and 2 g of water in the 25 mL beaker. The mixture was sonicated at 24 kHz, 100 W using an ultrasonic system (UP201S with Sonotrode S3, Hielscher Ultrasonics GmbH, Teltow, Germany) for 15 and 30 min with a pulsing cycle of 30 s on/30 s off. The experiment was carried out at 130 °C and room temperature (RT: 25 °C). For the recovery process, 40 mL of deionized water and 40 mL of acetone were added to the mixture in a 100-mL flask and stirred at 250 rpm for 1 h. Centrifugation at 6000 rpm for 10 min was employed to remove the supernatant, and the recovered biopolymer was washed with deionized water (3 times) to eradicate IL and freeze-dried for 48 h^29^. The pretreated biomass was subjected to enzymatic hydrolysis and further study.

### Compositional analysis

In the pretreated and untreated wheat straws, hemicellulose and cellulose content, along with lignin, including acid-insoluble and acid-soluble lignin, were obtained in line with NREL standard procedures^41^. The sugar content of the biomass was measured using the Well Chrom 2000 HPLC system (Knauer company) equipped with a Refractive Index (RI) K-2501 detector employing a Nucleosil-100 NH_2_ column, with acetonitrile and water 80:20 representing the mobile phase at a flow rate of 1 mL/min.

### Characteristics of untreated and pretreated biomass

#### X-ray diffraction (XRD)

This study used a PW 3710 Philips diffractometer equipped with the radiation of Co Kα (λ = 1.78 A°) at 30 mA and 40 kV to obtain XRD patterns of the untreated and US/IL-pretreated wheat strawsThese samples were scanned in the range of 5°–90° (2θ) with a step size of 0.02. The peak-height method was used to calculate the crystallinity index from the PXRD data through the Eq. 1^34,42,43^:

1 $$ {\text{CrI }}\left( \% \right) \, = {\text{ I}}_{{00{2}}} {-}{\text{ I}}_{{{\text{am}}}} /{\text{I}}_{{00{2}}} \times {1}00 $$

where I_002_ is the maximum intensity of the (002) plane, and I_am_ is the minimum intensity between the (101) and (002) planes according to the Segal method^30,33,42^.

#### Fourier-transform infrared (FTIR)

A Bruker Spectrometer Vector 33 was applied to determine the FTIR spectra of the untreated and treated wheat straws at a wave number ranging between 4000–400 cm^−1^ with a resolution of 4 cm^−1^ and 45 scans per sample. The preparation of the samples was done by mixing biomass with KBr and pressing.

#### Scanning electron microscopy (SEM)

The surface morphologies of the untreated and IL-pretreated wheat straws were closely analyzed by scanning electron microscopy (S360 CAMBRIDGE). To prepare the samples for SEM, they were treated as per the procedure described here: They were first kept in a solution containing 2% glutaraldehyde and 0.1 M phosphate buffer (pH = 7.2) at 4 °C overnight before being washed three times with phosphate buffer, followed by dehydration of the samples in ethanol concentrations of 50%, 70%, 90%, 96%, and 100%. Each step was incubated for 10 min. After that, the gold-coating process was applied to turn the samples conductive^33,34^.

### Enzymatic saccharification

US/IL pretreated and untreated wheat straws were exposed to enzymatic saccharification with 5% biomass in a sodium citrate buffer of 0.05 M (pH 4.8) with 0.2% sodium azide to prevent contamination. The cellulase enzyme was added at a loading of 28 FPU/g of biomass. The reaction was conducted in a shaker incubator for 72 h at a temperature of 50 °C with 200 rpm agitation. The samples were obtained at 0, 6, 24, 48, and 72 h and boiled at 90 °C for 5 min to stop enzyme activity. After centrifugation at 10,000 rpm for 5 min, the concentration of Total Reducing Sugar (TRS) was measured by the 3,5-dinitrosalicylic acid (DNS) method^45^ using a UV/VIS TG80 + Spectrophotometer. The glucose and xylose contents were obtained by HPLC. All analyses were carried out in duplicate. The saccharification yield was calculated as the ratio of the amount of glucose and xylose produced by enzymatic hydrolysis to the concentration of the aforementioned sugars present in the recovered biomass following IL-US pretreatment^29^.

### Batch fermentation studies

The fermentability of enzymatic hydrolysate was evaluated by the fermentation of the hydrolysates by *S. cerevisiae* (PTCC 5052) made available by IROST, Iran. The hydrolysates obtained through the saccharification step were segregated through centrifugation from the solid fraction. Because sodium azide represents a metabolite inhibitor, a distinct set of enzymatic hydrolyses, which had been utilized for fermentation, were carried out regardless of sodium azide and sampling. The glucose content of the hydrolysates was measured and concentrated to reach 100 g/L through vacuum evaporation at 50 °C, enriched with the necessary nutrients^45^. The media were inoculated with *S. cerevisiae* preculture*,* which was cultured in the YPD medium, with 3 g of yeast cells (on a dry weight basis) per liter of cellulosic hydrolysate (equivalent to OD_600_ 5) and incubated at 30 °C for 96 h. Fermentations were carried out in serum bottles (volume 10 mL) with rubber stoppers on which syringe needles were impinged to release the produced CO_2_. At regular 24-h intervals (0, 24, 48, 72, and 96 h), ethanol concentration was determined by YL6500 GC which was supplied by a TRB-G27 column and FID detector at 220 °C employing helium as the carrier gas.

## Results and discussion

### Effect of IL-US pretreatment on compositional analysis

Compositional analyses of both pre- and untreated straws were performed to assess the capability of IL-US pretreatment for biomass dissolution. The results of compositional analysis and the amount of recovered biomass from untreated and pretreated wheat straws are highlighted in Table 1. As mentioned in this table, the chemical composition of wheat straw before pretreatment comprised 35.7% glucan, 29.7% hemicellulose, 18.8% lignin, and 6.1% ash, which is consistent with other reported values^2,9^. The remaining mass, up to 100%, is probably related to lipids, proteins, and other compounds in the wheat straw^46^. Among different pretreatment conditions, the pretreated wheat straw at a temperature of 130 °C for 30 min demonstrated the highest dissolution yield compared to other pretreated samples (pretreated at a temperature of 130 °C for 15 min and pretreated at room temperature for 15 and 30 min).

**Table 1:** Chemical composition of both wheat straws.

Composition analysis, wt.%										
Pretreatment	Time of treatment (min)	Glucan	Xylan	Arabinan	Lignin		Total lignin	delignification	Ash	Recovered solid
					ASL	AIL				
Untreated		35.7 ± 0.3	26.1 ± 0.3	3.5 ± 0.1	7.2 ± 0.2	11.6 ± 0.4	18.8 ± 0.6	–	6.1 ± 0.2	100
IL-US Pretreated at RT	15	39.4 ± 0.4 *p* = *0.001*	23.6 ± 0.2	3.2 ± 0.1	5.5 ± 0.2	5.8 ± 0.3	11.3 ± 0.5	39.7	7.2 ± 0.4	80.2
	30	39.7 ± 0.3 p = 0.001	22.4 ± 0.2	2.8 ± 0.1	5.5 ± 0.1	5.7 ± 0.4	11.2 ± 0.5	40.3	7.5 ± 0.3	79.9
IL-US Pretreated at 130 °C	15	45.7 ± 0.4 p = 0.0001	15.7 ± 0.1	-	3.7 ± 0.1	2.4 ± 0.2	6.0 ± 0.4	67.8	10.2 ± 0.5	56.0
	30	53.9 ± 0.4 p = 0.0001	14.2 ± 0.1	-	3.8 ± 0.1	0.9 ± 0.1	4.7 ± 0.1	74.9	11.3 ± 0.8	45.2

ASL: Acid soluble lignin.

AIL: Acid insoluble lignin.

It provided excellent delignification up to 75%, 52.2% removal of hemicellulose, and a 51.0% increase in the glucan content, although the pretreatment of wheat straw with the same conditions for 15 min significantly affected the removal of hemicellulose and lignin in comparison to the untreated one. As listed in Table 1 glucan analysis shows that the time of treatment as well as the treatment process temperature are important for releasing glucans from biomass. P-values support the preceding statement by demonstrating that the mean glucan differs between treatments.

As reported by Smuga-Kogut et al.^47^, the optimum temperature for wheat straw fractionation is 130 °C. The outcome showed that the two pretreated samples had greater cellulose content, ranging between 28 and 51%, implying that hemicellulose and lignin in these samples were separated. However, the quantity of glucan in the biomass did not significantly vary in the pretreatment. It is suggested that some of the glucans in wheat straw be added to the hemicellulose and exist in the form of mixed linkage glucans so that near-quantitative cellulose recovery in the biomass can be obtained^21^. Given the amorphous morphological characteristics of hemicellulose, monomerization becomes much more convenient, compared to that of cellulose. It has been reported that hemicellulose removal would enhance cellulose’s enzymatic saccharification^43^. Eradication of hemicellulose augments the microfibril surfaces of biomass, promotes the enzymes’ contact with cellulose, and finally attenuates unnecessary binding of cellulase and hemicelluloses, which enhances the enzymatic saccharification of cellulose^48^. In addition, this pretreatment method results in significant delignification at 1:5 g/g biomass loading, which is an integral part of lignocellulosic fractionation for successful enzymatic saccharification^48^. It should be noted that the application of a high biomass loading significantly affects the cost of the biorefining process through a reduction in the size of the reactor required.

In comparison, the recovered biomass after pretreatment at 130 °C for 15 and 30 min decreased by 45% and 56%, respectively, suggesting that these conditions have a significant effect on the fractionation of cellulose microfibrils. A compositional analysis of the pretreated biomass illustrates that the insertion of hemicellulose and lignin into the IL solution represents a justified cause for the mass loss with respect to the prime biomass weight.

It was observed that in the case of pretreatment at room temperature, both of the pretreated samples exhibited almost similar results in removing lignin (~ 40%) and recovering biomass (~ 80%), which were much lower than the data attained in pretreatment at 130 °C. Based on these results, temperature has a critical effect on the dissolution of biomass, which could be attributed to the reduced recalcitrance of biomass at high temperatures and higher energy input^49^. Parallel findings are demonstrated for biomass pretreatment using different ILs and other pretreatment technologies, which showed enhancement of biomass fractionation upon a rise in the temperature through the course of pretreatment^20,50,51^.

The data of these pretreatments in comparison with those obtained in the same study^48^ using the heat treatment of wheat straw with ([TEA][HSO_4_]) at 130 °C for 30 min revealed that ultrasound irradiation enhanced the efficiency of pretreatment and delignification. Therefore, heat treatment in IL for 30 min at 130 °C resulted in the increase of 4.8% glucan and the removal of 18.6% hemicellulose and 45.4% lignin, while the use of ultrasound irradiation at 100w-24 kHz at the same time led to a 52% increase in glucan and a 54.3% decrease in hemicellulose and 74.9% of lignin, which is much higher than heat pretreatment alone. The effectiveness of the ultrasound techniques in improving the pretreatment effectiveness could be associated with the mechano-acoustic effects of ultrasound. This technique is known to enhance mass transfer, which results in increased biomass availability in the following processes^35^. Using ultrasound technology in combination with different pretreatment technologies leads to a high delignification efficiency of lignocellulosic biomass^52,53^.

These results are consistent with those obtained by Montalbo-Lomboy^29^, who reported that in the treatment of switchgrass with a conventional heat treatment ([Bmim][Cl]) at 130 °C, 12 to 24 h are required for fractionation of the biomass. Moreover, 53% removal of lignin was observed after 24 h of treatment, while using ultrasound irradiation with an amplitude of 160 μm and a frequency of 20 kHz for 4 min managed to eliminate 50.8% of lignin. Therefore, the use of ultrasonication shortens treatment time significantly and achieves optimal results. Moreover, Chen et al.^54^ indicated that a combination of US and [Amim][Cl] pretreatment resulted in enhancement of the effectiveness of pretreated pulp fibers with regard to increasing the glucan content and decreasing the hemicellulose and lignin levels. It was also reported that 190 min was required for cellulose to completely dissolve in [C_4_mim][Cl], whereas the total time was reduced to 60 min by applying ultrasound irradiation for 20 min^55^. Ultrasound irradiation also increased porosity in cellulose fiber, and breaking the α-O-4 or β-O-4 bond in the lignin resulted in a decrease in pretreatment time^34^. Moreover, the same findings regarding [TEA][HSO_4_] IL application to biomass dissolution were found in the related literature. At 130 °C, 3-h pretreated wheat straw with [TEA][HSO_4_] resulted in 80% delignification and 64.4% hemicellulose removal, highlighting the effectiveness of [TEA][HSO_4_] pretreatment in the biomass dissolution^48^. The pretreatment of four agricultural feedstocks (wheat straw, sugarcane bagasse, rice husk, and rice straw) with [TEA][HSO_4_] and 20 wt% H_2_O at a biomass-to-solvent ratio of 1:10 g/g demonstrated about 82% dissolution of lignin with the pretreatment process, generating significantly digestible cellulose-rich pulps^56^. Based on the fractionation effectiveness of pretreated *Miscanthus* with [TEA][HSO_4_] at 1:10 biomass loading, about 85% lignin and 100% hemicellulose elimination were observed at a temperature of 120 °C for 24 h^21^. Moreover, [TEA][HSO_4_] was reportedly recovered by more than 99%, owing to the IL’s non-volatile nature.

One important property of ILs that makes them attractive for pretreatment purposes is their recyclability. The recycling capacity of the IL can help reduce the cost of the pretreatment. The low volatility of IL makes it feasible for recycling studies ^26^.

Furthermore, [TEA][HSO_4_] solution is recycled to a greater degree for more treatment batches; thus, the effectiveness of fractionation from the viewpoint of lignin extraction is maintained every time^21^. A low concentration of protic ionic liquids (PILs) was designed for fractionation of coir and pith by using [TEA][HSO4]. A solid recovery of 71.87% and 68.9% were obtained for pretreated coir and pith, respectively. The glucose content in the treated coir and pith increased by 1.11 and 1.21-fold after pretreating, respectively. The recovery and reusability studies showed the recycled PIL could be utilized three times. Further application of low concentrations of PIL suggests the possibility of a new process design for a biorefinery to achieve low operating costs^57^.

### The effects of IL-US pretreatment on wheat straw structure

#### XRD analysis

**Figure 1:**
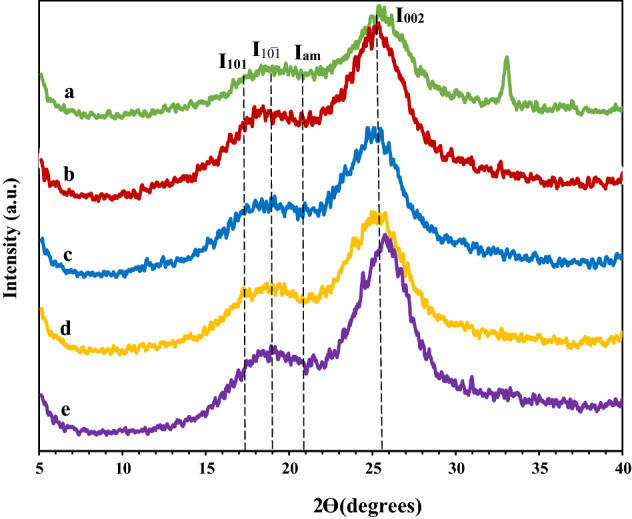
X-ray diffractograms of the untreated and treated wheat straws (**a**: untreated, **b**: at RT-15 min, **c**: at RT-30 min; **d**: at 130 °C-15 min; **e**: at 130 °C-30 min).

Cellulose crystallinity is considered a determining factor in the efficiency of enzymatic saccharification^33,40,59,60^. CrI can be altered due to cleavage of H-bond in the cellulose chain resulting from different pretreatment methods^43,58,60^. As the XRD patterns are shown in Fig. 1, the Segal equation was employed for calculating the CrI of samples. In the XRD patterns, the major peaks of (101), (10̅1), and (002) lattices are associated with the planes of crystalline cellulose I polymorph^34,43^. As listed in Table 2, CrI values for all the US-IL pretreated samples at room temperature and 130 °C exhibited no or little changes. In both US-IL pretreated samples, at room temperature for 15 and 30 min, CrI values decreased from 68 to 59% and 63%, respectively, while the pretreated samples at 130 °C (for 15 and 30 min) had CrI values almost similar to the untreated samples, which were 66% and 71%, respectively. The data exhibited no important alteration in CrI of the pretreated samples in association with the untreated ones. However, the dissolution of biomass and removal of lignin and hemicellulose occurred in pretreated samples, suggesting that removal of amorphous parts and peeling reactions in this area probably happened in the pretreatment process^30,32^. Moreover, the reported studies suggest that amorphous cellulose breaks down significantly at lower pHs and that it, therefore, leads to an increase in CrI under acidic conditions^60,61^. These data are consistent with the reported ones in the literature where the values of CrI for the pretreated samples increased in some cases. The same results were achieved in the pretreated samples using various ammonium hydrogen sulfate ILs, where the CrI of the pretreated samples increased^22^. It seems appropriate to consider that the acidic ILs and severe pretreatment conditions at high temperature increase the crystallinity, as was also reported by Morais^62^. Furthermore, in the pretreated samples with US-IL pretreatment, CrI increased from 19.5% in the untreated water hyacinth to 32.4% in the treated samples. These findings show that applications of ultrasonic irradiation can enhance the deconstruction of the crystalline cellulose in US-IL pretreatment^32^. Likewise, in pretreated *Eucalyptus* using ultrasound-IL and ultrasound-alkaline pretreatment, both samples experienced an increase in CrI compared to untreated biomass (44.8% and 47.6%, respectively, vs. 40.8%)^30^.

**Table 2:** Crystallinity index of both wheat straws.

Samples	Time of treatment (min)	Crystallinity Index (%)	LOI of wheat straw (%)	TCI of wheat straw (%)
Untreated		68	4.9	0.12
IL-US Pretreated at RT	15	59	4.0	0.16
	30	63	3.6	0.16
IL-US Pretreated at 130 °C	15	66	3.4	0.19
	30	71	3.3	0.18

Lignocellulose is a complex macromolecule with an amorphous lignin and hemicellulose matrix and crystalline cellulose. Therefore, it is clear that its crystallinity can be altered as it undergoes chemical and physical treatment, removing a major portion of lignin and some hemicellulose. During pretreatment, the inter- and intramolecular hydrogen bonds between glucose monomers in cellulose chains change. Therefore, a challenging strategy towards determining the crystallinity after treatment exists. This is due to the fact that some parts of lignin, hemicellulose, and distorted cellulose remain in the treated biomass^62^. The increase in CrI values of pretreated samples can be attributed to the strong removal of the amorphous fraction during pretreatment. It is conceivable that temperature promotes the increase in CrI, which can also be confirmed by the increasing intensity of 2θ signal found at 22.2°^62^ or removal of lignin or hemicellulose from biomass and the transformation of crystalline cellulose to an amorphous state^57^.

Consequently, in comparison with RT-pretreated samples for 15 and 30 min, the pretreated samples at 130 °C pretreated for 30 min exhibit a higher CrI value.

#### FTIR analysis

FTIR analysis was conducted for untreated and pretreated wheat straws to further characterize the chemical structural modifications due to US-assisted IL pretreatment,.

**Figure 2:**
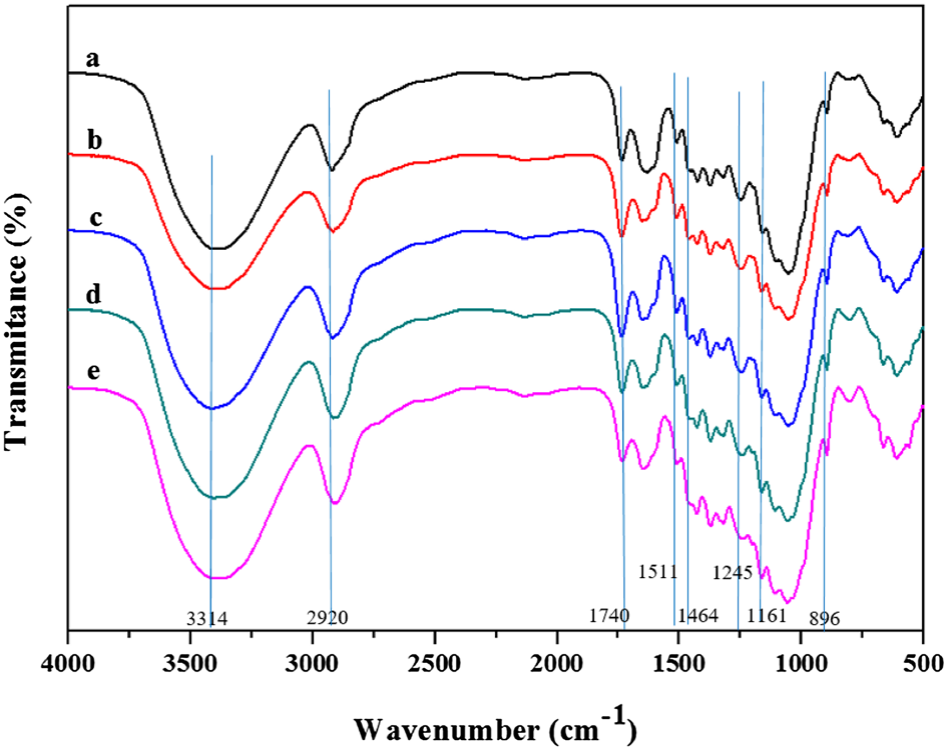
FTIR spectra of both wheat straws (**a**: untreated, **b**: at RT-15 min, **c**: at RT-30 min; **d**: at 130 °C-15 min; **e**: at 130 °C-30 min).

From the FTIR spectra shown in Fig. 2, the intensity of some peaks was either weaker or stronger in the pretreated samples than in their untreated counterparts, which were related to cellulose, hemicellulose, and lignin.

The peaks at 1511 cm^−1^ (aromatic skeleton C–C stretching in lignin) and 1464 cm^−1^ (asymmetric bending in CH_3_ in lignin) related to the lignin decreased significantly for the pretreated biomass at 130 °C; in particular, the peaks at 1464 cm^−1^ were removed from the pretreated biomass in 30 min (or no absorption peak at 1464 cm^−1^ was detected in the pretreated biomass for 30 min) compared to the untreated wheat straw. Moreover, a decline in the intensity of the peak at 1245 cm^−1^ (C–O vibration in the syringyl ring in lignin) was observed in all the pretreated biomasses, but a reduction in the biomass in pretreated samples at 130 °C was greater for 30 min, which was established by the composition analysis. It is suggested that violent delignification occurs during US-IL pretreatment at 130 °C, while a slight one may arise from other pretreatments, which is in line with outcomes from composition analysis. Moreover, the intensity of the peak at 1320 cm^−1^ (syringyl and guaiacyl condensed lignin) decreased in both of the pretreated samples at room temperature, which pointed to the occurrence of lignin removal^11,33,60^.

The characteristic cellulose/hemicellulose peaks at 1245 cm^−1^ (C–O stretching in lignin and hemicellulose), 1053 cm^−1^ (C–O stretching in cellulose and hemicellulose), and 1740 cm^−1^ (carbonyl groups from hemicellulose) became weaker for all the pretreated samples, particularly for 130 °C pretreated samples due to hemicellulose removal^30,60^. Based on the obtained results, it was determined that throughout the course of US-IL treatment, deacetylation took place when the FTIR absorption peak was at 1740 cm^−1^. Due to the bond disintegration, enzymatic hydrolysis is improved, representing an obstacle to the lignocellulosic biomass dissolution in hemicellulose^30^. The composition evaluation results, shown in Table 1, attest to the attenuation of the hemicellulose fraction throughout the pretreatment process. In addition, cellulose characteristic bands were determined in all of the pretreat samples in regions 1161, 1110, and 1035 cm^−1^^9^.

The TCI (Total Crystallinity Index; A_1368_/A_2900_) and LOI (lateral Order Index; A_1424_/A_896_) values of the samples were calculated based on the spectra referred to as CrI^63^. The values of TCI and LOI are shown in Table 2. In all of the pretreated samples, the LOI value decreased, ranging from 18.4% to 32.6%, while the TCI value increased, as mentioned in Section "XRD analysis". Altogether, FTIR attested to the observations that the IL pretreatment in conjunction with ultrasound irradiation might effectively disrupt the crystalline and ordered structure of cellulose in the wheat straw.

The amorphous cellulose peaks at 896 cm^−1^ were sharp and strong in the two pretreated biomass samples at 130 °C. They underwent a minor augmentation in the pretreated samples at RT for 30 min and had the same intensity in the untreated samples as in the pretreated samples for 15 min. However, the peaks at 1424 cm^−1^ (CH_2_ scissor motion in cellulose) were linked to the breaking of the intramolecular hydrogen bond in the samples’ -CH_2_-OH . These peaks represent the lateral order index or crystallinity^11,60^ that experienced a decrease in all of the pretreated samples. However, the peaks at 1373 cm^−1^ and 2920 cm^−1^ were correlated with C–H bending vibration in cellulose and hemicellulose, and CH and CH_2_ stretching increased in all of the pretreated biomass samples that corresponded to total crystallinity^11,33^.

#### SEM analysis

**Figure 3:**
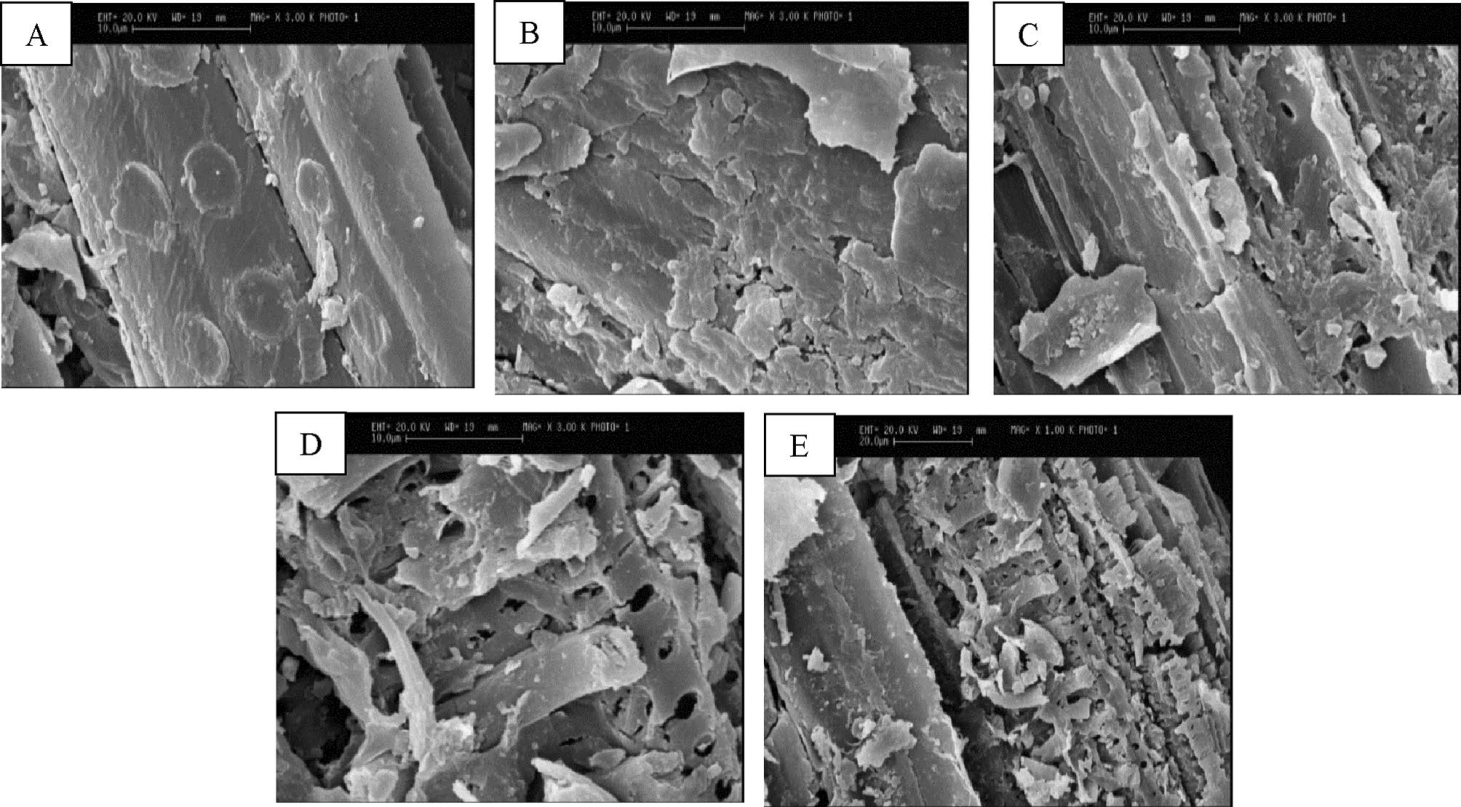
SEM of untreated wheat straw (**A**), IL-US pretreated at RT for 15 min (**B**), and 30 min (**C**), IL-US pretreated at 130 °C for 15 min (**D**), and 30 min (**E**).

Figure 3 displays SEM micrographs of untreated (A), IL-US pretreated at room temperature for 15 min (B) and 30 min (C), IL-US pretreated at 130 °C for 15 min (D), and 30 min (E) wheat straw. Clearly, the untreated wheat straw surface structure is highly ordered, intact, and rigid, which is due to the microcrystalline structure of cellulose covered by lignin and hemicellulose that prevents the accessibility of enzymes to cellulose, while the surface of IL-US pretreated biomass is rougher, disordered, and agglomerated. It is clear that these variations in the surface of pretreated biomass could be associated with biomass cell wall destruction over the dissolution of biomass and removal of hemicellulose and lignin from the structure, as confirmed by compositional analysis and FTIR. Moreover, large pores and cracks found on the surface of the pretreated biomass could be attributed to the cavitation effect of ultrasound irradiation. The majority of changes observed on the surface of samples were related to both pretreated biomass at 130 °C, where numerous penetrations and porosities were observed on the surface, and the completely changed structure, which was common in the ultrasonically treated samples, resulting in an increase in cellulase enzyme accessibility and enhancement of enzymatic saccharification. Enzymatic saccharification is significantly affected by the approachability of enzymes to cellulose. The conducted morphological investigations point to a considerable rise in the pores and the biomass surface upon pretreatment, leading to enhanced enzymatic hydrolysis^29,30^.

### Effect of pretreatment with IL-US on enzymatic hydrolysis

Enzymatic saccharification was performed for 72 h at 50 °C using a commercial cellulase from *T. reesei* at a loading of 28 FPU/g biomass to further investigate the effects of ultrasound irradiation-assisted, cost-effective ionic liquid on the dissolution and fractionation of wheat straw.

**Figure 4:**
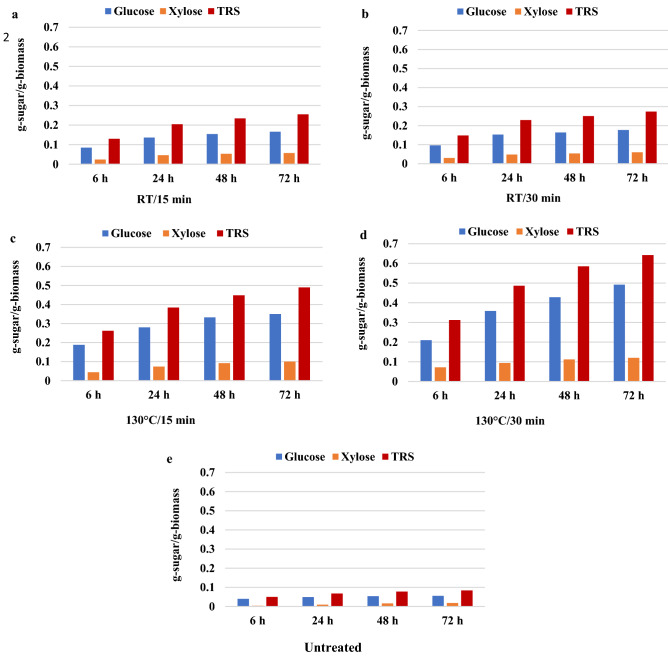
TRS, glucose, and xylose production of untreated and pretreated wheat straws (**a**: at RT-15 min, **b**: at RT-30 min; **c**: at 130 °C-15 min; **d**: at 130 °C-30 min; **e**: untreated).

The time profile of the TRS as well as glucose and xylose production in the enzymatic saccharification of different pretreated wheat straw samples at a solid loading of 20% is shown in Fig. 4a–d. As shown in the figure, without pretreatment (Fig. 4e), the amount of production of the total reducing sugar is 4.2 mg/mL (0.08 g-sugar/g-biomass) after 72 h. In contrast, with US-IL pretreatment at room temperature, this increased to about 13 mg/mL (0.26 g-sugar/g-biomass), regardless of the pretreatment time. Pretreated wheat straw samples at 130 °C produced a higher sugar yield of 24.5 mg/mL (0.49 g-sugar/g-biomass) in 15 min and 32.1 mg/mL (0.64 g-sugar/g-biomass) in 30 min. The pretreatment of wheat straw in [TEA][HSO_4_] using the conventional heating method at 130 °C for 30 min resulted in TRS production of 16.9 mg/mL (0.34 g-sugar/g-biomass), which was lower than that in the case of US-IL pretreatment^48^. Based on the above findings, the drawback of using heat pretreatment in IL can be overcome by combining IL and ultrasonic waves, i.e., an approach that might enhance enzymatic hydrolysis within a shorter time frame. The amount of produced glucose in enzymatic saccharification from RT- pretreated samples in 15 and 30 min and 130 °C in pretreated samples in 15 and 30 min were 8.3 g/L (0.17 g-sugar/g-biomass), 8.9 g/L (0.18 g-sugar/g-biomass), 17.5 g/L (0.35 g-sugar/g-biomass) and 24.6 g/L (0.49 g-sugar/g-biomass), respectively (Fig. 4a–d). The amount of produced xylose in enzymatic saccharification in the above conditions was 2.9 g/L (0.06 g-sugar/g-biomass), 3.0 g/L (0.06 g-sugar/g-biomass), 5 g/L (0.1 g-sugar/g-biomass), and 6 g/L (0.12 g-sugar/g-biomass), respectively (Fig. 4a–d), while it was 2.8 g/L (0.06 g-sugar/g-biomass) for glucose and 0.9 g/L (0.02 g-sugar/g-biomass )for xylose in untreated wheat straw (Fig. 4e).

**Figure 5:**
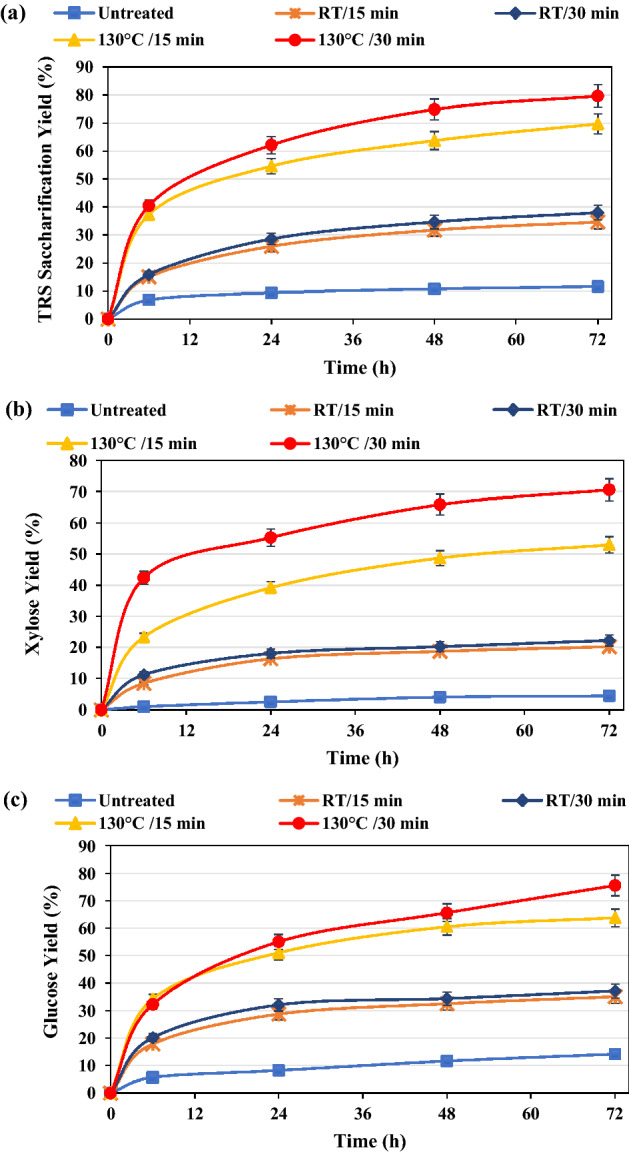
Saccharification yields of TRS (**a**), glucose (**b**), and xylose (**c**) of both wheat straws.

The glucose, xylose, and TRS saccharification yields of the untreated and pretreated wheat straws are given in Fig. 5. The effect of temperature on the TRS saccharification yield of pretreated biomasses revealed that the yields at room temperature were 34.6 and 38.0% for the pretreated sample in 15 and 30 min, respectively; the yields at 130 °C were 67.7% in 15 min and 79.8% in 30 min, while it was 11.6% for the untreated wheat straw. Furthermore, without IL pretreatment, the glucose saccharification yield was almost 14.1%; it increased to 35.1% and 37.2% in RT pretreated samples at 15 and 30 min, respectively, and to 63.7% in pretreated samples for 15 min at 130 °C; besides, with further pretreatment for 30 min, it increased to 75.6%.

The maximum xylose yield of 70.6% was achieved in 30 min for the pretreated sample at 130 °C, while it was lower than 5% in the case of untreated wheat straw and 53.0% for the 15-min pretreated sample at the same temperature. Therefore, the achievement of high yields of glucose and xylose saccharification by applying inexpensive IL coupled with US pretreatment and a commercial enzyme is remarkable, which reveals the high effectiveness of this approach in the biorefinery process.

Future bulk prices of conventional dialkylimidazolium ionic liquids are estimated to be $40–81/kg, when compared to the techno-economic analysis of the bulk-scale synthesis of 1-methylimidazolium hydrogen sulfate, which is about $2.96–5.88/kg, indicates a very promising reduction of ILs cost to as low as common organic solvents such as acetone and toluene^21^. This is very promising, as triethylammonium hydrogen sulfate water mixtures can be made available at bulk scale for as little as $1.24/kg. The synthesis of this low-cost IL also enjoys several other advantages. It is easy to synthesize in very few steps and needs no purification process. It also has a lower impact on the environment by reducing waste byproducts, solvent losses, energy uses, and therefore less CO_2_ generation^22^. The above-mentioned advantages are supported by the fact that low-cost ILs are also recyclable, which adds to the value of their utilization as a solvent for lignocellulose pretreatment. Usually the solvent cost determines how much solvent loss and purge can be tolerated before process economics are excessively impacted^64^. Despite this important criteria, only a limited number of studies have investigated the reuse of ILs after pretreatment to date.

According to the achieved data, the commercial enzyme used is highly capable of converting hemicelluloses to xylose, which in turn produces a notable saccharification yield. This will be beneficial for the cost-effectiveness of bioprocesses when a microorganism utilizing xylose is used in the fermentation process, which produces industrially important metabolites.

Moreover, from the obtained data, it has been concluded that temperature has a noteworthy effect on IL pretreatment for successful enzymatic saccharification^20,50^.

The comparable saccharification yields were obtained based on the findings existing in the published works employing inexpensive ILs for pretreatment. The saccharification yields of 77% and 45% were reported with [TEA][HSO_4_] pretreatment of *Miscanthus giganteus*^20^ and switchgrass *(Panicum virgatum*), respectively^22^ using Cellic® CTec_2_ enzyme. After treatment of wheat straw, rice straw, and sugarcane bagasse at 170 °C with [TEA][HSO_4_] for 30–45 min, glucose yields approached 90% in enzymatic hydrolysis using the Cellic® CTec_2_ enzyme^48^. A glucose yield of 50.36% was obtained after 0.5 h of pretreatment of wheat straw with [TEA][HSO_4_], and following more efforts into pretreatment for 3 h, the yield reached 87.19% after 72 h of enzymatic hydrolysis using commercial cellulase. The 3-h pretreated sample experienced a maximum xylose yield of 78.23%, while it was lower than 5% in untreated wheat straw and 30.93% for the 0.5-h pretreated one^48^. Moreover, the findings of this study demonstrate that the IL-US pretreatment has a meaningful impact on carbohydrate generation during enzymatic saccharification compared to the stirring and heating processes in the conventional thermal pretreatment. It was reported that the sugar yield of thermally pretreated bamboo powder in cholinium IL (choline acetate) at 110 °C for 60 min was about 55%, while after ultrasonic pretreatment in IL at 25 °C for 60 min, 92% of cellulose hydrolysis to glucose^34^ was achieved. Moreover, sonication at a frequency of 20 kHz with an amplitude of 160 µm for the treatment of switchgrass in ([Bmim][Cl] led to a shorter pretreatment time. A 100% yield of glucose digestibility was observed after 3-h enzymatic saccharification of 24-h pretreated biomass with IL at 130 °C, while this result was achieved after 4 min of pretreatment with a combination of IL-US^29^. Yu et al.^28^ investigated the enzymatic hydrolysis of sugarcane bagasse and wheat straw pretreated with a combination of ultrasonic frequencies (20–50 kHz) and [Bmim] Cl and [Bmim] AOc at 80 °C for 30 min. The optimum yields of glucose, measured at 40.3% for sugarcane bagasse and 53.2% for wheat straw were achieved at 20 kHz with [Bmim][Cl]. Furthermore, it was reported that the optimal ultrasonic frequency led to the maximum yield of reduced sugars, which varied in terms of IL type, biomass nature, and hydrolysis process^28^. Moreover, it was demonstrated that the application of IL combined with ultrasonic treatment enhanced saccharification yield by about 1.7 times. A fractionation of rice straw was investigated with US and different forms of ILs^18^. The TRS yield attained from the pretreated rice straw with choline hydroxide and ultrasonic irradiation (300 W) at a frequency of 40 kHz was 96.2% in 4 h, which was about 20% more than the value found when ultrasonic radiation was not used. Morphological analyses of the biomass have shown that US treatment with [C][OH] effectively separates lignin from biomass and destroys their hard structure, leading to increased cellulose accessibility to the enzyme’s molecules^18^. Moreover, ultrasonic-assisted pretreatment could increase cellulose conversion due to improvements in morphological degradation and chemical structure^31^. Therefore, ultrasonic-assisted IL pretreatment is a viable and effective alternative to lignocellulosic biomass.

The enzymatic saccharification yield with ultrasound was 30% greater than that without irradiation in the same condition^48^. These findings remarkably emphasize the effect of ultrasound irradiation on reducing pretreatment time, which considerably affects the capital cost of the process and the economic aspect of bio-refining. This significant decrease in pretreatment time at amplified biomass loading without lessening the yield is promising for the development of this pretreatment process. In addition, ease of ultrasound installation and operation using environmentally acceptable technology without producing any chemicals (toxic or harmful byproducts) combined with low-cost IL as a green solvent constitutes the advantages of applying the results of the present study. In conclusion, ultrasound technology has gained significant attention and is used in the pretreatment of lignocellulosic biomass to increase saccharification and ethanol production yields due to high delignification in a quicker pretreatment process.

Based on the obtained results, upon increasing the pretreatment time, the saccharification yield increased slightly (less than 12%). Therefore, from an economic point of view, it may be concluded that 15 min of pretreatment is adequate for appropriate dissolution of wheat straw to achieve high yields in saccharification and fermentation processes, while a long pretreatment time leads to an insignificant improvement of subsequent processes.

### Effect of IL-US pretreatment on Fermentation

**Figure 6:**
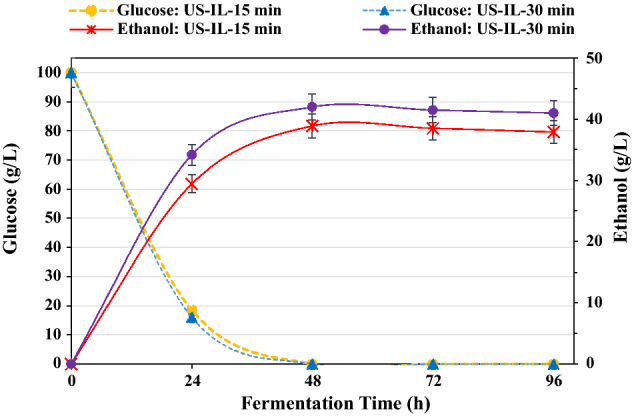
The time taken for the consumption of glucose and ethanol generated throughout fermentation.

Fermentation of cellulosic hydrolysates of two pretreated wheat straws with ultrasound-[TEA][HSO_4_] at 130 °C was performed using *S. cerevisiae.* Based on the results of the composition analysis of the samples and enzymatic hydrolysis, it was observed that the two pretreated wheat straws with ultrasonic-assisted IL at room temperature, as mentioned in sections "Effect of IL-US Pretreatment on Compositional Analysis" and "Effect of pretreatment with IL-US on enzymatic hydrolysis", did not show a significant performance; therefore, the fermentation process was not carried out for these two samples. The time taken for consumption of glucose and ethanol generated throughout the 96-h fermentation is shown in Fig. 6. As shown in this figure, ethanol production in pretreated samples for 15 and 30 min at 130 °C reached 38.9 and 42.0 g/L, respectively, which corresponded to productivity rates of 0.8 and 0.9 g/L/h. However, the amount of ethanol produced from untreated wheat straw was only 5.5 g/L.

The initial glucose concentration in the fermentation medium was 100 g/L Glucose was completely consumed within 48 h of fermentation; after that, not only was ethanol production increased to a higher yield, but it was also slightly reduced over time. The ethanol yields for pretreated wheat straw at 15 and 30 min were 76.1 and 82.2% of the theoretical yield, respectively.

The fermentation results obtained in this study are consistent with previous findings using other lignocellulosic biomass and different pretreatment technologies. The ethanol yields of 81.5% and 66.8% of theoretical yield were achieved with [EMIM][OAc] pretreated spruce powder and chips, and yields of 81% and 51.8% with [BMIM][OAc] pretreated spruce powder and chips at 120 °C for 15 h, while yields of untreated spruce powder and chips were 9.7% and 2.7%, respectively^65^. Also, [EMIM][OAc] pretreatment of aspen wood for 1 to 5 h at 120 °C resulted in ethanol yields ranging from 73.5 to 81.2% of theoretical yield^45^. Moreover, [EMIM][OAc] pretreatment of *Guinea* grass at 157 °C for 30 min resulted in an ethanol yield of 81.9% in a 24-h fermentation^50^. Besides, an ethanol yield of 70% was detected in *eucalyptus dunnii* bark pretreated with [BMIM][Cl] at a temperature of 140 °C for 8 h in the SHF optimization^66^.

### Mass balance

**Figure 7:**
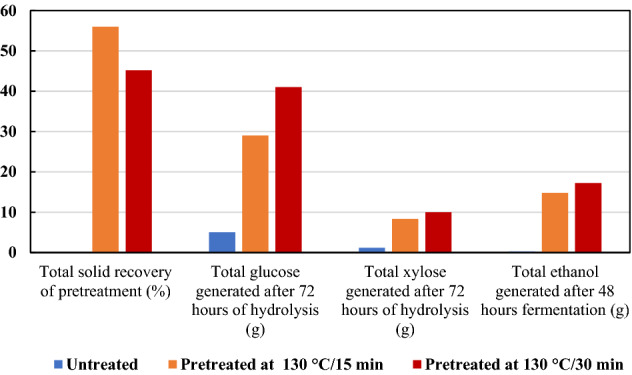
Summary of the overall mass balance for pretreatments, enzymatic hydrolysis, and fermentation of wheat straw (all the obtained values are in grams per 100 g of initial wheat straw).

The overall mass balance for the economic assessment of bioethanol generation is of high importance. Figure 7. highlights the mass balance of three key steps of the process, such as pretreatment, hydrolysis, and fermentation, for untreated and pretreated samples. The recovered biomass was 56.0 and 45.2% in the case of 15- and 30-min pretreatment at 130 °C, respectively. For every gram of initial wheat straw, 0.3 and 0.4 g of glucose might be approximately generated upon 15- and 30-min pretreatment with [TEA][HSO_4_] in 72 h of saccharification, respectively. This would roughly result in the production of 148.1 and 172.5 g ethanol from 1 kg of the initial wheat straw pretreated with IL/US in 15 and 30 min at 130 °C, respectively, which corresponds to 1 L of ethanol from 6.7 and 4.6 kg of wheat straw, while there was merely 2.7 g ethanol per kg of the untreated wheat straw.

## Conclusion

This work pointed to the promising and viable applicability of a pretreatment method by employing US combined with inexpensive IL [TEA][HSO_4_] for wheat straw dissolution at room temperature and 130 °C for 15 and 30 min of pretreatment, respectively. This research achieved high saccharification and ethanol yields in a short pretreatment time at high biomass loading and emphasized the remarkable role of ultrasound irradiation in reducing pretreatment time, which significantly affected the capital cost of the process and the economic aspect of bio-refining. In addition, applying commercial cellulase to enzymatic hydrolysis ensured high yields, thus suggesting the feasibility of this method on an industrial scale. Another research area for future study in IL pretreatment technology that can offer a better techno-economical characterization of the intended process is the recycling of IL, which must be considered along with lowering the cost and loading of IL. In conclusion, this approach could be an efficient and economically viable alternative to conventional pretreatment techniques by overcoming the high cost of the process and shortening the associated time duration so as to bring about the industrial development and viability of US-assisted IL technology in biorefinery and biofuel generation. The findings have promising implications for developing a low-cost IL method, aided by the US, for the conversion of biomass straw (cellulose) to ethanol.

## Data avilibility

The raw datasets used and analyzed during the current study are not publicly available but are available from the corresponding authors upon request.
